# A Coordinated Approach to Perioperative Rehabilitation to Enhance Outcomes in Melanoma: Protocol for the CARE-Melanoma Pilot Randomized Controlled Trial

**DOI:** 10.2196/90946

**Published:** 2026-08-21

**Authors:** Jenna Smith-Turchyn, Jordon L Hvizd, Valerie Francescutti

**Affiliations:** 1 School of Rehabilitation Science McMaster University Hamilton, ON Canada

**Keywords:** melanoma, rehabilitation, physical activity, prehabilitation, physical function, pilot trial

## Abstract

**Background:**

Rehabilitation is beneficial for individuals with cancer across all phases of care, but access remains limited for many survivors, including individuals with melanoma. As new treatment strategies for melanoma are introduced, there is an opportunity to assess perioperative rehabilitation strategies for patients undergoing surgery during and after neoadjuvant treatment.

**Objective:**

This paper describes the protocol for a 2-armed pilot randomized controlled trial with the overall objective of determining the feasibility and preliminary effects of a perioperative rehabilitation strategy for individuals with melanoma at a single cancer institution in Ontario, Canada.

**Methods:**

In total, 30 participants, including English-speaking adults with a current melanoma diagnosis scheduled to undergo surgery after neoadjuvant immunotherapy, will be randomized 1:1 to either the intervention or control group. The intervention will include 4 perioperative intervention sessions (2 sessions before surgery and 2 sessions after surgery) led by a trained physiotherapist or kinesiologist. Participants in the intervention group will receive education on the benefits of exercise; how to exercise safely; and rehabilitation techniques to maximize range of motion, strength, and function. Participants will also receive a tailored exercise program and will establish goals and action plans at each session. The control group will receive usual care (no rehabilitation). The primary outcome is feasibility (measured via recruitment, retention, and adherence rates). Secondary outcomes will be collected before and after the intervention and include overall impairment score (measured via the Edmonton Symptom Assessment Scale), physical activity level (Godin Leisure Time Exercise Questionnaire), functional mobility (6-minute walk test and 30-second sit-to-stand test), range of motion (goniometry), grip strength (handheld dynamometry), quality of life (Functional Assessment of Cancer Therapy-Melanoma), perception of health status (EQ-5D-3L), and medication use. Feasibility will be analyzed using descriptive statistics (mean [SDs] or frequency [%]), as appropriate. Secondary outcomes will be reported using means, SD, and CIs of outcomes. Cohen *d* effect sizes will be calculated and reported for each secondary outcome to inform a future full-scale trial. STATA/MP (version 14) will be used for all statistical analysis, with significance set at *P*<.05.

**Results:**

Trial enrollment began in June 2026, and 3 participants have been recruited to date. Recruitment is expected to be completed by December 2026. Results will be submitted for publication by mid or late 2027.

**Conclusions:**

This pilot trial aims to address the evidence gap on the feasibility and preliminary effects of rehabilitation for individuals with melanoma, laying the groundwork for a larger randomized controlled trial and future implementation of rehabilitation for this population. By implementing rehabilitation around the surgical phase, we aspire to enhance patient outcomes by maximizing function and reducing side effects, facilitating a faster return to meaningful daily activities.

**Trial Registration:**

ClinicalTrials.gov NCT07320222; https://clinicaltrials.gov/study/NCT07320222

**International Registered Report Identifier (IRRID):**

PRR1-10.2196/90946

## Introduction

### Background

Melanoma, a serious form of skin cancer, has become increasingly prevalent in Canada [1]. In recent years, Canadians have become increasingly aware of the importance of early detection and treatment [1]. However, surgical intervention for melanoma, while often necessary for effective management, can result in significant physical and emotional challenges for patients [2,3]. In Canada, the treatment landscape for melanoma is evolving with the introduction of new neoadjuvant immunotherapy regimens aimed at improving patient outcomes for those with more advanced melanoma [4,5]. Recent advancements include the use of combination therapies that integrate immune checkpoint inhibitors with targeted therapies, which are designed to enhance tumor response before surgical resection [4]. These innovative approaches aim to reduce tumor size and potentially improve the rate of complete surgical removal [4]. Ongoing clinical trials continue to evaluate the effectiveness and safety of these regimens, reflecting a commitment to optimizing preoperative treatment strategies and improving outcomes for those with melanoma [6]. Neoadjuvant immunotherapy is commonly administered over 6 weeks after the initial consultation for ipilimumab-nivolumab combination therapy or over 12 weeks after the initial consultation for pembrolizumab monotherapy. While these advances offer promising improvements in disease management, the introduction of immunotherapy during the preoperative period may also increase treatment burden. Patients may experience the combined effects of surgery and immunotherapy-related toxicities, including fatigue, reduced muscle strength, and impaired physical function. These potential consequences should be considered and proactively addressed alongside these changes in medical management.

Rehabilitation plays a crucial role in the recovery process for individuals with cancer, helping individuals regain strength, range of motion (ROM), mobility, and confidence [7-9]. Comprehensive rehabilitation programs can support not only physical healing but also address psychological concerns, enabling patients to better navigate their journey toward recovery and improved quality of life [7-10]. Exercise is one component of rehabilitation and has demonstrated improvements in fitness levels, strength, physical function, fatigue, and quality of life in those with cancer [7,8,10]. Current exercise guidelines for those with cancer include weekly aerobic, resistance, and flexibility training [7,11]. Aerobic exercise can help improve cardiovascular fitness, while resistance and functional exercise support improvements in strength, mobility, and performance of daily activities [7].

With the new presurgery immunotherapy treatment approach being implemented in Canada for individuals with melanoma, we now have more time to implement perioperative rehabilitation (rehabilitation around the surgical procedure) for this patient group. In individuals with other forms of cancer, prehabilitation, which is defined as a proactive approach to health care that involves enhancing a patient’s physical and psychological well-being before a surgical procedure [12], has been found to offer significant benefit by enhancing ROM, strength, physical function, and overall health prior to surgery or treatment [12-15]. By engaging in targeted rehabilitation before surgery, patients may improve their ROM, strength, endurance, and physical outcomes, leading to optimization of function and reduced treatment-related side effects after treatment [13-15]. Additionally, as we have demonstrated in our previous cancer rehabilitation research [16], rehabilitation before surgery can help optimize functional outcomes and alleviate anxiety and improve psychological well-being, empowering patients to approach their survivorships phases with confidence and resilience [17-19]. Despite growing evidence supporting rehabilitation interventions across multiple cancer populations, individuals with melanoma remain underrepresented in rehabilitation research.

Little evidence exists to guide rehabilitation delivery throughout prehabilitation and early postoperative phases in melanoma care, and no standardized rehabilitation models have been developed for patients undergoing this new treatment approach. Rehabilitation is not provided routinely for individuals with melanoma globally [20], and no form of perioperative rehabilitation services are available for most individuals with melanoma. Therefore, the purpose of this pilot trial is to determine the feasibility and preliminary effects of a rehabilitation strategy for individuals with melanoma before and after surgery within a cancer institution in Canada. The present intervention is informed by established principles in cancer rehabilitation [7,8] and prehabilitation [12,13] and was adapted from our previous cancer rehabilitation research demonstrating the feasibility and benefits of physiotherapy-led exercise and self-management interventions in oncology populations [16]. This was done because it targets impairments that are common across cancer populations, such as fatigue, physical deconditioning, and reduced functional capacity and quality of life. However, modifications were made to account for the unique treatment considerations experienced by individuals with melanoma, including timing of neoadjuvant immunotherapy, impairments related to wide local excision and node dissection, mobility concerns, and postoperative pain. The individualized approach used in the current intervention allows recommendations to be tailored according to treatment-related and melanoma-specific functional impairments. The selected feasibility outcomes will inform implementation of a future definitive trial specifically in those with melanoma, and the selected secondary outcomes reflect the hypothesized effects of the intervention.

### Research Questions and Hypotheses

This trial will answer 3 research questions.

First, is perioperative rehabilitation feasible for individuals diagnosed with melanoma requiring neoadjuvant immunotherapy and then receiving surgery? We hypothesize that the intervention will be feasible, as demonstrated by recruitment, retention, and adherence rates of >70% and high patient satisfaction with the intervention.

Second, do individuals with melanoma who participate in perioperative rehabilitation have improved outcomes (improved overall impairment score, physical activity level, functional mobility, ROM, grip strength, quality of life, perception of health status, and medication use) compared to those receiving usual care (no rehabilitation)? We hypothesize that those participating in the intervention will have improved overall impairment score, physical activity level, functional mobility, ROM, grip strength, quality of life, perception of health status, and medication use, compared to those receiving usual care.

Third, what is the frequency and nature of adverse events associated with perioperative rehabilitation for individuals with melanoma? We hypothesize that perioperative rehabilitation for individuals with melanoma will be safe and associated with few, if any, intervention-related adverse events.

## Methods

### Study Design

**Figure 1:**
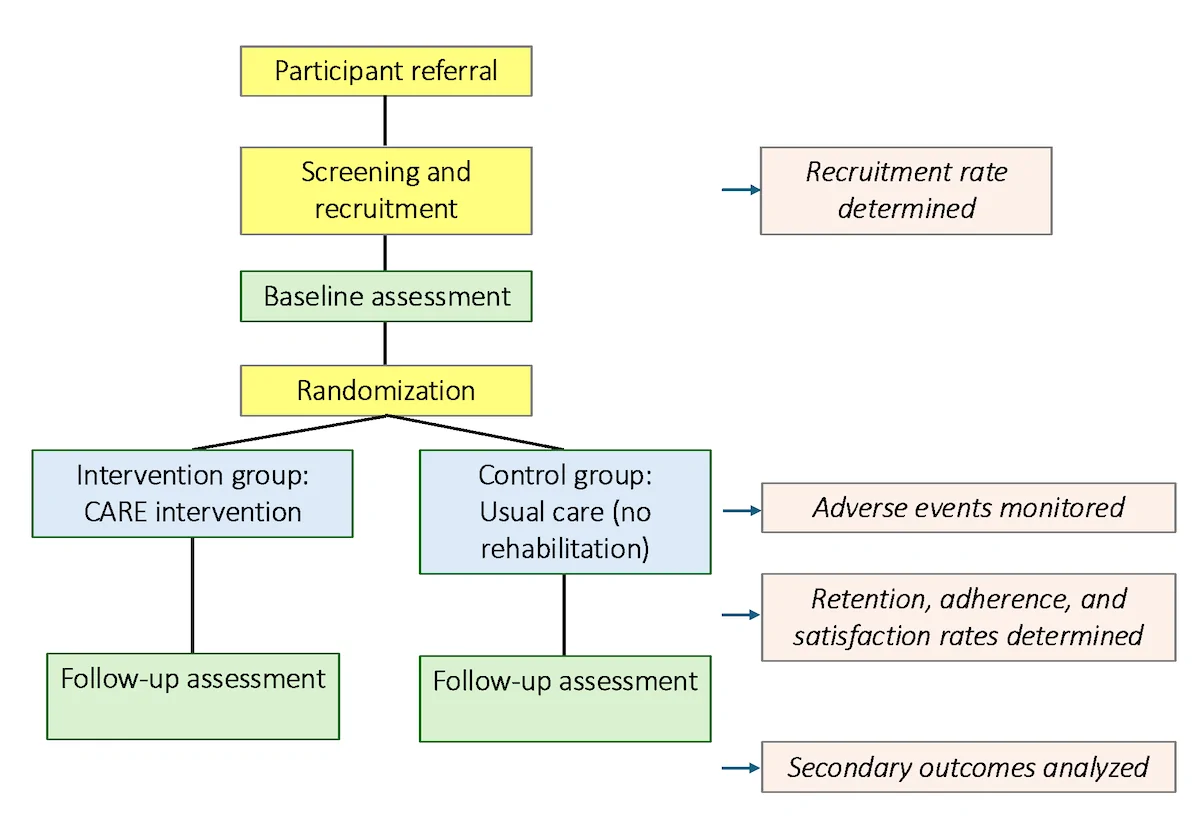
Study flow.

This is a pilot, parallel group randomized controlled trial (RCT). It will use a 1:1 allocation ratio, and participants will be randomly assigned to either the intervention or control group. Outcome assessments will be conducted at baseline and postintervention time points with assessors and data analysts blinded to group allocation. The protocol adheres to the SPIRIT (Standard Protocol Items: Recommendations for Interventional Trials) 2025 checklist [21] for protocols of randomized trials. Figure 1 shows the flow of participants across the study and associated analysis time points.

### Ethical Considerations

This study was approved by the Hamilton Integrated Research Ethics Board (19542) and is registered on ClinicalTrials.gov (NCT07320222). All participants will complete written informed consent prior to taking part in the study. Participant privacy and confidentiality will be protected throughout the study, with outcome data being deidentified and no identifying information included in study reports. Study data will be stored securely and accessed only by authorized members of the research team. Participants will be reimbursed for parking expenses incurred while attending in-person intervention and assessment sessions. No additional compensation is provided.

### Participants and Recruitment

Eligible participants for this study include individuals who (1) have a diagnosis of melanoma, (2) are English-speaking, (3) are adults aged 18 years or older, and (4) are scheduled to undergo surgery at the Juravinski Cancer Centre in Hamilton, Ontario, after receiving a neoadjuvant immunotherapy regimen. Eligible potential participants will be referred to the study by their surgical oncologist or oncology care team using a consent to contact form, which includes the participants contact information and has the referring physician sign off that they are safe to exercise. Contact information of those who sign the consent to contact form will be given to the study research coordinator. They will be further screened for eligibility via their preferred method of communication by the research coordinator. Participants will be excluded if they (1) are aged <18 years, (2) are not undergoing neoadjuvant immunotherapy, and (3) self-report any chronic condition, cognitive impairment, or injury that would prevent them from participating independently in moderate intensity exercise.

### Sample Size

Based on sample size calculations for pilot data [22,23], we expect to recruit 30 participants for the study (15 in each group). For this project, the sample size was calculated based on the expected proportion of success of the primary feasibility outcome [23]. Using a Z value corresponding to a 95% CI (1.96), a desired margin of error (E) of 0.2, and an estimated population proportion of success (p) of 0.75 (based on expected estimated adherence rates), the calculated sample size for this pilot study was 18 participants (9 per group). With an expected dropout rate of up to 30% [24], the final sample size for this project was set at 30 participants (15 per group) to accommodate 1:1 block randomization and provide a conservative buffer.

### Procedure

Once eligibility is confirmed, participants will complete a baseline assessment, after which they will be divided into 2 groups: intervention (CARE intervention) or control (usual care).

#### Intervention Group

The intervention group (CARE intervention) will include 4 perioperative intervention sessions led by a physiotherapist or kinesiologist trained in the treatment of individuals with cancer. The total intervention time will be approximately 4 months, with 2 of the intervention sessions occurring prior to surgery and 2 occurring after surgery. The intervention was designed to coincide with the immunotherapy treatment regime and key perioperative milestones [20]. The 2 preoperative sessions provide an opportunity for assessment, exercise education, prehabilitation, and development or an individualized exercise plan. They will be conducted in person whenever participants visit the cancer center for a scheduled appointment. The postoperative sessions support reassessment, progression of rehabilitation, management or postsurgical issues, and transition to long-term self-management. Participants will have the option of taking part in the postsurgery sessions in person or virtually. The approximately 4-month duration was selected to encompass the presurgical and early postsurgical recovery period while maintaining feasibility and minimizing participant burden in this pilot trial.

**Figure 2:**
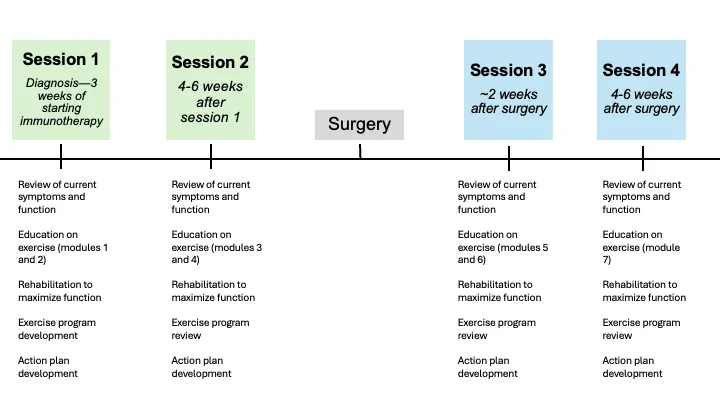
CARE intervention components.

Each intervention session will include five components based on our previous research and guidelines for survivors of cancer: (1) review of current symptoms and function, (2) educational modules on exercise viewed on an iPad with the trainer, (3) tailored rehabilitation to maximize function, (4) home exercise program development or review, and (5) action plan development for participants to work on between sessions. Table 1 describes each intervention component. Figure 2 provides an overview of the CARE intervention components across the study period.

**Table 1:** Components of the CARE intervention.

Component	Description
Review of current symptoms and function	Sessions 1-4 (items checked) Current symptoms (eg, pain, fatigue, range of motion, strength, balance scan) Posture Functional concerns (mobility observation) Vital signs (eg, heart rate, blood pressure, SpO2) Other changes since last visit
Education on exercise [16]	Session 1 Module 1: benefits of exercise, exercise safety, how to self-manage, how to set goals (15-minute video module) Module 2: posture, breathing techniques for stress and anxiety, managing energy levels, aerobic exercise description and parameters (15-minute video module) Session 2 Module 3: Strength training and parameters (15-minute video module) Module 4: Flexibility and alternative exercise types and parameters (15-minute video module) Session 3 Module 5: Self-monitoring activity level and function (15-minute video module) Module 6: Communicating about exercise, recovery after surgery (15-minute video module) Session 4 Module 7: How to progress and move forward with exercise, how to modify to meet needs (30-minute video module)
Tailored rehabilitation to maximize function	Sessions 1-4 (as needed by individual) Joint mobility to improve range of motion and muscle tension Postural awareness Balance Mobility
Home exercise program development/review^a^ [7]	Sessions 1-4 (following general exercise guidelines for cancer survivors [7,11]) Aerobic exercise (up to 150 minutes of moderate intensity aerobic exercise) Resistance training (twice weekly strength training for all major muscle groups) Flexibility exercises most days of the week for all major muscle groups
Action plan development [25]	Sessions 1-4: participants develop action plans related to exercise to work on between sessions. These include the following: Reflection of previous action plan success (if applicable) Current plan details (what, how, where, when, and how often) Rating of importance (0-10 scale) and confidence (0-10 scale) Potential barriers and how to adapt or modify the plan Potential supports Back-up plan Progress plan Commitment statement

^a^Trainers will tailor specific exercise program based on participant and cancer specific characteristics and work up to achieving these guidelines.

#### Control Group

The control group will receive usual care, which in the context of melanoma care at the Juravinski Cancer Centre includes no form of rehabilitation. Participants assigned to this group will not receive a rehabilitation referral from the study team; however, they are not limited from discussing rehabilitation options with their medical oncologist if they wish to learn more. Participants are not restricted from accessing rehabilitation services outside of the study.

### Outcomes and Measurement Instruments

Outcomes will be assessed before and after the intervention by a trained assessor blinded to group allocation and will be documented on REDCap (Vanderbilt University; a secure online research application for managing research data). Assessments will be scheduled to coincide with participants’ cancer center appointments to improve accessibility for all patients.

#### Primary Outcome: Feasibility (Research Question 1)

##### Overview

The primary outcome of this pilot trial will be feasibility of the intervention. Feasibility will be measured by recruitment, retention, adherence rates, and participant satisfaction. Recommendations for progression to a full-scale trial will be based on feasibility data and published recommendations for progression criteria [26-28]. Table 2 outlines feasibility components and interpretation or progression criteria.

**Table 2:** Feasibility components and progression criteria [26,28].

Feasibility criteria	Go: proceed to RCT^a^	Amend: proceed with changes	Stop: do not proceed unless changes are possible
Patient recruitment	If >70% of eligible potential participants are recruited to take part	If 50% to 70% of eligible potential participants are recruited to take part	If <50% of eligible potential participants are recruited to take part
Participant retention	If >70% of enrolled participants are retained until study completion	If 50% to 70% of enrolled participants are retained until study completion	If <50% of enrolled participants are retained until study completion
Participant adherence	If participants attend >70% of intervention sessions	If participants attend 50% to 70% of intervention sessions	If participants attend <50% of intervention sessions
Participant satisfaction	If each of the 4 AIM^b^ questions has a median response score of ≥4 out of 5	If each of the 4 AIM questions has a median response score of 3 out of 5	If each of the 4 AIM questions has a median response score of ≤2 out of 5

^a^RCT: randomized controlled trial.

^b^AIM: Acceptability of Intervention Measure.

##### Recruitment Rate

Recruitment rate for this study is defined as the percentage of eligible patients referred to the study who agree to participate.

##### Retention Rate

Retention rate is the percentage of recruited and enrolled participants who are retained until study completion.

##### Adherence Rate

Adherence rate will be measured as adherence to the intervention session and is defined as the number of sessions attended.

##### Participant Satisfaction

Participant satisfaction will be measured using the Acceptability of Intervention Measure [29]. This measure assesses 4 components of satisfaction on a 5-point Likert scale ranging from 1 (completely disagree) to 5 (completely agree) [29]. Higher scores represent higher levels of intervention acceptability and satisfaction [29].

#### Secondary Outcomes (Research Question 2)

Secondary outcomes to be assessed include an overall impairment score, physical activity level, functional mobility, ROM, grip strength, quality of life, perception of health status, and medication use.

#### Overall Impairment Score

The Edmonton Symptom Assessment Scale (ESAS) [30] will be used to measure overall impairment. The ESAS is a self-report measure designed to assess the severity of various symptoms commonly experienced by those with cancer [30]. It consists of 8 symptoms (pain, fatigue, drowsiness, nausea, appetite, shortness of breath, depression, and anxiety), which are each rated on a scale of 0 (no symptoms) to 10 (worst possible symptoms), along with a question on overall well-being at a single point in time (rated from 0 [best well-being] to 10 [worst possible well-being]) [30]. Higher scores indicate more severe symptoms and higher overall symptom burden [30]. The ESAS has good reliability and validity among survivors of cancer [31].

#### Physical Activity Level

The Godin Leisure Time Exercise Questionnaire [32] will be used to assess physical activity level. It is a self-report tool designed to assess an individual’s level of physical activity over a 7-day period [33]. Respondents report the frequency of mild, moderate, and vigorous physical activity they engage in for at least 15 minutes during a typical week. Higher scores indicate a greater level of physical activity [33]. A score of 24 or more indicates “sufficiently active” or meeting current exercise guidelines, while lower scores suggest “insufficient activity” [32,33]. This measure has demonstrated high levels of reliability and validity in survivors of cancer [34].

#### Functional Mobility

Functional ability will be assessed using the 30-second sit-to-stand (30sSTS) test [35] and the 6-minute walk test (6MWT) [36]. Standardized instructions will be provided by the assessor before each test, and participants will receive assessor supervision and support throughout administration of these tests. The 30sSTS is a simple assessment of functional ability in which participants begin by sitting in a chair with their arms crossed over their chest. Participants rise to a standing position and then return to a seated position, repeating the movement as many times as possible within 30 seconds. The number of completed repetitions is counted as the score, with higher scores representing higher levels of functional mobility. Normative values exist by age and sex [37]. Reliability and validity studies have shown this test to be reliable and valid when assessing physical performance in survivors of cancer [38]. The 6MWT has the participant walk as far as possible in 6 minutes along a flat, straight corridor. Participants are encouraged to walk at a comfortable pace and can pause or use an assistive device if necessary. Outcomes are interpreted as the total distanced walked (meters) in 6 minutes. Normative values exist based on age and sex [39]. The 6MWT has demonstrated high reliability and validity in survivors of cancer [40].

#### ROM

ROM will be assessed using goniometry. Goniometry is the gold standard for ROM measurement [41]. Based on the location of the participants’ melanoma surgery and lymph node dissection (ie, neck, axillary, or groin), ROM assessments will be conducted for the upper extremities (UEs; if neck or axillary surgery) or lower extremities (LEs; if groin surgery). For UE ROM, movements to be assessed include shoulder flexion, abduction, internal rotation, and external rotation; elbow flexion and extension; and wrist flexion, extension, radial deviation, and ulnar deviation. For LE ROM, movements to be assessed include hip flexion, extension, internal rotation, and external rotation; knee flexion and extension; and ankle plantar flexion and dorsiflexion. Normative values exist for each joint, with higher values representing better ROM [41].

#### Grip Strength

Handheld dynamometry [42] will be used to measure grip strength. For this test, individuals will sit in a comfortable position with their elbow flexed at 90° and their wrist in a neutral position while exerting maximal force by squeezing the handheld device. The test will be repeated twice for each hand, and the average grip strength will be calculated and recorded in kilograms. Normative values exist for age and sex, and higher scores indicate greater muscle strength [42]. Handheld dynamometry exhibits good reliability and validity in survivors of cancer [43].

#### Quality of Life

The Functional Assessment of Cancer Therapy-Melanoma (FACT-M) [44] will be used to assess quality of life. This self-report measure includes a series of items that evaluate various domains of health-related quality of life, including physical well-being, social or family well-being, emotional well-being, functional well-being, and melanoma-specific concerns. Each item is rated on a Likert scale ranging from “not at all” to “very much.” Outcomes can be interpreted based on both subscale and total score, with higher scores indicating better quality of life. Reliability and validity studies support the FACT-M’s use among those with cancer [45].

#### Perception of Health Status

The EQ-5D-3L [46] will be used to measure health status. It encompasses 5 dimensions: mobility, self-care, usual activities, pain, and anxiety or depression; respondents rate the severity of each dimension as having no problems, some problems, or extreme problems. It also includes a visual analog scale where respondents will rate their overall health on a scale of 0 (worst imaginable health) to 100 (best imaginable health). Higher scores indicate better health status. Research has demonstrated its reliability and validity among survivors of cancer [46].

#### Medication Use

Medication use will be collected using a self-report questionnaire. The questionnaire will ask participants to list the medications they are currently using and have used 7 days prior to the assessment (drug name and dosage). After the surgery, the questionnaire will also ask if they have taken specific medication since surgery (acetaminophen, antiinflammatories, muscle relaxants, neuropathic pain medications, or narcotics), how long they took the medication after the surgery, and the dosage. Tracking medication will allow us to identify changes in medications that may occur during the perioperative period, explore whether medication use acts as a potential confounder when interpreting intervention-related change, and inform the design and covariate selection for a future larger-scale trial.

#### Adverse Events (Research Question 3)

The safety of our intervention will be assessed by monitoring adverse events throughout the study period. Participants will be asked to report any adverse event at each study contact. They will be classified by type, severity, duration, and relatedness to the intervention based on the National Cancer Institute Common Terminology Criteria for Adverse Events [47]. Serious adverse events will be reported to the Hamilton Integrated Research Ethics Board in accordance with institutional policies. The trial will be suspended if one or more serious adverse events are deemed definitely or probably related to the intervention by the study team and research ethics board.

### Data Analysis Plan

Feasibility outcomes (research question 1; recruitment, retention, adherence, and satisfaction rates) will be analyzed using descriptive statistics (mean [SD] or frequency [%]), as appropriate. Preliminary estimates of effects *(*research question 2*)* will be reported using mean (SDs) and CIs of outcomes for continuous outcomes. Given the pilot nature of this trial and the limited sample size, Cohen *d* effect sizes will be calculated and reported for each secondary outcome. Effect sizes will be interpreted alongside CIs to provide estimates of the magnitude of effect and to inform a future full-scale trial. Medication use will be summarized descriptively for each treatment group. The proportion of participants using each medication class will be reporting using frequencies (%s), while duration of medication use and dosage will be summarized using mean (SDs) or median (IQRs), as appropriate. The frequency and nature of adverse events (research question 3) will be summarized descriptively. An intention-to-treat analysis will be conducted, with missing data handled using multiple imputation. STATA/MP (version 14; StrataCorp) will be used for all statistical analyses, with significance set at *P*<.05.

### End Points

The intervention will take place over 4 months, with assessments taking place before and after the intervention. Therefore, all data are expected to be collected within 9 months. Data analysis will then be conducted and is expected to take approximately 1 month.

## Results

This study is supported by the Hamilton Health Sciences (HHS) Research and HHS Foundation through the HHS Breakthrough Awards program, with funding awarded in November 2025. Enrollment began in June 2026, and 3 participants have been recruited to date. Recruitment is expected to be completed by December 2026. Results are expected to be submitted for publication by mid or late 2027. Table 3 shows the expected timeline of study progress.

**Table 3:** Study timeline.

	2026							2027								
Activity	Jul	Aug	Sep	Oct	Nov	Dec	Jan		Feb	Mar	Apr	May	Jun	Jul	Aug	Sep
Recruitment	✓	✓	✓	✓	✓	✓										
Baseline assessments	✓	✓	✓	✓	✓	✓	✓									
Intervention period	✓	✓	✓	✓	✓	✓	✓		✓	✓	✓	✓				
Follow-up assessments				✓	✓	✓	✓		✓	✓	✓	✓				
Analysis													✓	✓		
Manuscript preparation and journal submission														✓	✓	✓

## Discussion

### Overview

Rehabilitation has been shown to benefit individuals with cancer across all phases of the cancer trajectory [7,8,10,48]. However, rehabilitation remains inaccessible to many survivors of cancer [20,49]. With evolving management strategies for individuals with melanoma and the introduction of new neoadjuvant immunotherapy regimens aimed at improving outcomes [4,5], there is an opportunity to implement and evaluate perioperative rehabilitation strategies for this population. Although rehabilitation is well established in several cancer populations, evidence remains limited for those with melanoma, particularly those receiving neoadjuvant immunotherapy. The current study contributes to the emerging field of melanoma rehabilitation by assessing the feasibility of adapting established oncology rehabilitation interventions to a population with unique challenges. The intervention was designed to align with a new melanoma treatment pathway, incorporating individualized tailoring based on treatment status, symptom burden, functional limitations, and surgical recovery needs, acknowledging that those with melanoma may experience a combination of immunotherapy and surgery-related side effects requiring rehabilitation. Recognizing that treatment-related toxicities may affect study retention, several measures have been incorporated into this trial to minimize participant burden and maximize participation, including scheduling intervention visits alongside routine oncology appointments, offering virtual delivery of postoperative sessions, and tailoring rehabilitation recommendations to participants’ needs and functional status. Study retention and reasons for withdrawal, including those related to immunotherapy-associated adverse events, will be tracked as feasibility outcomes to inform future larger-scale trial design.

Overall, results of this pilot trial will help us determine the feasibility and preliminary effects of perioperative rehabilitation for individuals with melanoma who are scheduled to undergo surgery after neoadjuvant immunotherapy. These findings may inform the development of future melanoma-specific rehabilitation pathways and support the integration of rehabilitation services alongside evolving systemic treatment approaches. This work addresses an important gap in the literature and contributes to the expanding evidence base for exercise and rehabilitation across the cancer continuum.

### Strengths and Limitations

Strengths of this pilot trial include the unique implementation of a perioperative rehabilitation intervention for individuals with melanoma and the rigorous methodology used in this study. The results of this pilot trial will help determine whether the proposed intervention and recruitment strategies are feasible and can be implemented effectively in a larger trial in this setting. It will also provide valuable insights on resources required (such as time, personnel, and funding) for a larger trial. Furthermore, this trial will help us determine the satisfaction of participants, which can enhance implementation of future trials.

However, the findings of this project should be considered in the context of its limitations. First, as this is a pilot trial, the sample size is small and is not powered to detect definitive intervention effects. This limits power of the results explored for the secondary outcomes, and they should therefore be interpreted as preliminary. Furthermore, as recruitment occurred at a single site, generalizability of findings will be limited to the single site where this pilot trial is implemented, limiting external validity of the findings. Additionally, LE strength and lymphedema were not assessed in this study. Both factors may influence outcomes, specifically performance on the 6MWT and 30sSTS, and should be considered in future work. Furthermore, participants who choose to enroll may be more motivated to engage in rehabilitation than the broader melanoma population, and treatment-related effects may influence adherence and retention rates. Despite these limitations, this study addresses an important gap in the cancer rehabilitation literature.

### Future Direction

Although evidence supporting exercise and rehabilitation interventions continues to grow across oncology populations, little research has focused specifically on individuals with melanoma. This project will be the first step toward informing a full-scale RCT and supporting the future implementation of rehabilitation strategies for this population. Implementing perioperative rehabilitation has the potential to improve patient outcomes in both the short and long term. By maximizing functional capacity and minimizing treatment-related side effects early in the cancer care trajectory during the perioperative period, we aim to preserve function during subsequent treatment and facilitate an earlier return to meaningful activities at home and work for individuals with melanoma.

### Dissemination Plan

The knowledge translation (KT) plan aims to effectively disseminate study results and raise awareness about the potential for perioperative rehabilitation to improve outcomes in individuals with melanoma. Results will be disseminated locally by conducting “lunch and learn” sessions at local cancer centers and by having participants share “participant perspectives” with local cancer support organizations. In addition, a social media campaign will be implemented to extend the reach of study findings. This campaign will target individuals with cancer, supportive care organizations, and health professionals treating this population using platforms such as Bluesky, LinkedIn, X (formerly Twitter), and Facebook to share research highlights, infographics, and participant stories.

Traditional KT activities will also be incorporated to ensure that study findings are effectively disseminated to academic and professional audiences. A manuscript will be submitted to a peer-reviewed journal in oncology or cancer rehabilitation. Furthermore, results and practical recommendations will be presented at relevant conferences, allowing for engagement with clinicians, researchers, and policymakers. By combining these strategies, the KT plan aims to mobilize results to end users globally, ensuring that the evidence generated from this study informs best practices and enhances future support for individuals with melanoma.

## Data Availability

Data from this trial will be made available by the lead author (JS-T) upon reasonable request.

## Abbreviations

30sSTS: 30-second sit-to-stand
6MWT: 6-minute walk test
ESAS: Edmonton Symptom Assessment Scale
FACT-M: Functional Assessment of Cancer Therapy-Melanoma
HHS: Hamilton Health Sciences
KT: knowledge translation
LE: lower extremity
RCT: randomized controlled trial
ROM: range of motion
SPIRIT: Standard Protocol Items: Recommendations for Interventional Trials
UE: upper extremity

## References

[1] (2024). Canadian Cancer Statistics: a 2024 special report on the economic impact of cancer in Canada. Canadian Cancer Society.

[2] Surgery for melanoma skin cancer. American Cancer Society.

[3] Surgery for melanoma skin cancer. Canadian Cancer Society.

[4] Therien AD, Chime-Eze CM, Rhodin KE, Beasley GM (2024). Neoadjuvant therapy for melanoma: past, present, and future. Surg Oncol.

[5] Sun J, Kirichenko DA, Zager JS, Eroglu Z (2019). The emergence of neoadjuvant therapy in advanced melanoma. Melanoma Manag.

[6] Yuan F, Jing M, Chen X, Zhang X (2025). Effect of neoadjuvant therapy on survival outcomes in patients with melanoma: a systematic review and meta-analysis. EClinicalMedicine.

[7] Campbell KL, Winters-Stone KM, Wiskemann J, May AM, Schwartz AL, Courneya KS, Zucker DA, Matthews CE, Ligibel JA, Gerber LH, Morris GS, Patel AV, Hue TF, Perna FM, Schmitz KH (2019). Exercise guidelines for cancer survivors: consensus statement from International Multidisciplinary Roundtable. Med Sci Sports Exerc.

[8] Patel A, Friedenreich CM, Moore SC, Hayes SC, Silver JK, Campbell KL, Winters-Stone K, Gerber LH, George SM, Fulton JE, Denlinger C, Morris GS, Hue T, Schmitz KE, Matthews CE (2019). American College of Sports Medicine roundtable report on physical activity, sedentary behavior, and cancer prevention and control. Med Sci Sports Exerc.

[9] Schmidt ME, Wiskemann J, Ulrich CM, Schneeweiss A, Steindorf K (2017). Self-reported physical activity behavior of breast cancer survivors during and after adjuvant therapy: 12 months follow-up of two randomized exercise intervention trials. Acta Oncol.

[10] Cormie P, Zopf EM, Zhang X, Schmitz KH (2017). The impact of exercise on cancer mortality, recurrence, and treatment-related adverse effects. Epidemiol Rev.

[11] Segal R, Zwaal C, Green E, Tomasone JR, Loblaw A, Petrella T (2017). Exercise for people with cancer: a clinical practice guideline. Curr Oncol.

[12] Stout NL, Fu JB, Silver JK (2021). Prehabilitation is the gateway to better functional outcomes for individuals with cancer. J Cancer Rehabil.

[13] Saleh AM, Al Daragemeh AI, Abdel-Aziz HR (2024). Empowering wellness: a comprehensive narrative review of cancer prehabilitation from treatment onset to surveillance. Malays J Med Sci.

[14] Gennuso D, Baldelli A, Gigli L, Ruotolo I, Galeoto G, Gaburri D, Sellitto G (2024). Efficacy of prehabilitation in cancer patients: an RCTs systematic review with meta-analysis. BMC Cancer.

[15] Harris E, Marignol L (2024). Prehabilitation for patients with cancer undergoing radiation therapy: a scoping review. Clin Oncol (R Coll Radiol).

[16] Smith-Turchyn J, Stouth DW, Keogh JA, Ball E, Rodrigues M, Richardson J, Bordeleau L, Neil-Sztramko S, Levine O, Thabane L, Sabiston CM, Mukherjee SD (2025). Implementing exercise and self-management for women with breast cancer during treatment: results of the NEXT-BRCA randomized controlled trial. Sci Rep.

[17] Stout NL, Santa Mina D, Lyons KD, Robb K, Silver JK (2021). A systematic review of rehabilitation and exercise recommendations in oncology guidelines. CA Cancer J Clin.

[18] Chia Z, O'Brien M, Shortland J, Holmes HM, Giza D, Ngo-Huang A, Cheung KL, Parks RM (2025). The influence of physical post-operative rehabilitation interventions to improve upper limb strength in women undergoing breast cancer surgery: a systematic review of the literature. Eur J Surg Oncol.

[19] Olsson Möller U, Beck I, Rydén L, Malmström M (2019). A comprehensive approach to rehabilitation interventions following breast cancer treatment - a systematic review of systematic reviews. BMC Cancer.

[20] (2022). Skin cancer pathway map. Cancer Care Ontario.

[21] Chan AW, Tetzlaff JM, Altman DG, Laupacis A, Gøtzsche PC, Krleža-Jerić K, Hróbjartsson A, Mann H, Dickersin K, Berlin JA, Doré CJ, Parulekar WR, Summerskill WS, Groves T, Schulz KF, Sox HC, Rockhold FW, Rennie D, Moher D (2013). SPIRIT 2013 statement: defining standard protocol items for clinical trials. Ann Intern Med.

[22] Thabane L, Ma J, Chu R, Cheng J, Ismaila A, Rios LP, Robson R, Thabane M, Giangregorio L, Goldsmith CH (2010). A tutorial on pilot studies: the what, why and how. BMC Med Res Methodol.

[23] Hertzog MA (2008). Considerations in determining sample size for pilot studies. Res Nurs Health.

[24] Smith-Turchyn J, Richardson J, Tozer R, McNeely M, Thabane L (2020). Bridging the gap: incorporating exercise evidence into clinical practice in breast cancer care. Support Care Cancer.

[25] Lorig K, Holman H, McGowan PO, Sobel DS, Huebner TL, Laurent D, González V, Minor M (2013). Living a Healthy Life with Chronic Conditions: Self-Management of Heart Disease, Arthritis, Diabetes, Depression, Asthma, Bronchitis, Emphysema and Other Physical and Mental Health Conditions.

[26] Mellor K, Albury C, Dutton SJ, Eldridge S, Hopewell S (2023). Recommendations for progression criteria during external randomised pilot trial design, conduct, analysis and reporting. Pilot Feasibility Stud.

[27] Avery KN, Williamson PR, Gamble C, O'Connell Francischetto E, Metcalfe C, Davidson P, Williams H, Blazeby JM (2017). Informing efficient randomised controlled trials: exploration of challenges in developing progression criteria for internal pilot studies. BMJ Open.

[28] Herbert E, Julious SA, Goodacre S (2019). Progression criteria in trials with an internal pilot: an audit of publicly funded randomised controlled trials. Trials.

[29] Weiner BJ, Lewis CC, Stanick C, Powell BJ, Dorsey CN, Clary AS, Boynton MH, Halko H (2017). Psychometric assessment of three newly developed implementation outcome measures. Implement Sci.

[30] Hui D, Bruera E (2017). The Edmonton Symptom Assessment System 25 years later: past, present, and future developments. J Pain Symptom Manage.

[31] Richardson LA, Jones GW (2009). A review of the reliability and validity of the Edmonton Symptom Assessment System. Curr Oncol.

[32] Amireault S, Godin G (2015). The Godin-Shephard Leisure-Time Physical Activity Questionnaire: validity evidence supporting its use for classifying healthy adults into active and insufficiently active categories. Percept Mot Skills.

[33] Amireault S, Godin G, Lacombe J, Sabiston CM (2015). The use of the Godin-Shephard Leisure-Time Physical Activity Questionnaire in oncology research: a systematic review. BMC Med Res Methodol.

[34] Amireault S, Godin G, Lacombe J, Sabiston CM (2015). Validation of the Godin-Shephard Leisure-Time Physical Activity Questionnaire classification coding system using accelerometer assessment among breast cancer survivors. J Cancer Surviv.

[35] Díaz-Balboa E, González-Salvado V, Rodríguez-Romero B, Martínez-Monzonís A, Pedreira-Pérez M, Cuesta-Vargas AI, López-López R, González-Juanatey JR, Pena-Gil C (2022). Thirty-second sit-to-stand test as an alternative for estimating peak oxygen uptake and 6-min walking distance in women with breast cancer: a cross-sectional study. Support Care Cancer.

[36] Butland RJ, Pang J, Gross ER, Woodcock AA, Geddes DM (1982). Two-, six-, and 12-minute walking tests in respiratory disease. Br Med J (Clin Res Ed).

[37] Jones CJ, Rikli RE, Beam WC (1999). A 30-s chair-stand test as a measure of lower body strength in community-residing older adults. Res Q Exerc Sport.

[38] Blackwood J, Rybicki K (2021). Physical function measurement in older long-term cancer survivors. J Frailty Sarcopenia Falls.

[39] McKay MJ, Baldwin JN, Ferreira P, Simic M, Vanicek N, Burns J (2017). Reference values for developing responsive functional outcome measures across the lifespan. Neurology.

[40] Schmidt K, Vogt L, Thiel C, Jäger E, Banzer W (2013). Validity of the six-minute walk test in cancer patients. Int J Sports Med.

[41] Muir SW, Corea CL, Beaupre L (2010). Evaluating change in clinical status: reliability and measures of agreement for the assessment of glenohumeral range of motion. N Am J Sports Phys Ther.

[42] Jones DW, Robertson LD, Figoni SF (2009). A strength-endurance index for power grip. J Occup Rehabil.

[43] Pino-Ortega J, Carvajal-Espinoza R, Becerra-Patiño B, Gómez-Parra A, Moreno-Villanueva A (2025). Handgrip strength assessment in breast cancer patients: validity and reliability of the Activ5 dynamometer. Support Care Cancer.

[44] Lindqvist Bagge AS, Wesslau H, Cizek R, Holmberg CJ, Moncrieff M, Katsarelias D, Carlander A, Olofsson Bagge R (2022). Health-related quality of life using the FACT-M questionnaire in patients with malignant melanoma: a systematic review. Eur J Surg Oncol.

[45] Bharmal M, Fofana F, Barbosa CD, Williams P, Mahnke L, Marrel A, Schlichting M (2017). Psychometric properties of the FACT-M questionnaire in patients with Merkel cell carcinoma. Health Qual Life Outcomes.

[46] Kim SH, Jo MW, Lee JW, Lee HJ, Kim JK (2015). Validity and reliability of EQ-5D-3L for breast cancer patients in Korea. Health Qual Life Outcomes.

[47] (2017). Common Terminology Criteria for Adverse Events (CTCAE) version 5.0. U.S. Department of Health and Human Services.

[48] Cormie P, Atkinson M, Bucci L, Cust A, Eakin E, Hayes S, McCarthy AL, Murnane A, Patchell S, Adams D (2018). Clinical Oncology Society of Australia position statement on exercise in cancer care. Med J Aust.

[49] Fernandez S, Franklin J, Amlani N, DeMilleVille C, Lawson D, Smith J (2015). Physical activity and cancer: a cross-sectional study on the barriers and facilitators to exercise during cancer treatment. Can Oncol Nurs J.

